# Bilateral Sesamoiditis as First Manifestation of Gout

**DOI:** 10.1155/2020/8890549

**Published:** 2020-09-05

**Authors:** Daniel de Oliveira Beraldo, Sasha Duarte, Gustavo Pacheco, Rodrigo Barbosa, Carolina Mendes, Marcela Silva, Fabiana Beraldo, Andrei Alkmim, Ricardo Teixeira, Alexandre Bonfim

**Affiliations:** ^1^Hospital Renascentista, Pouso Alegre, MG, Brazil; ^2^Escola Paulista de Medicina-UNIFESP, São Paulo, SP, Brazil; ^3^Instituto do Coração-FMUSP, São Paulo, SP, Brazil

## Abstract

Sesamoiditis secondary to gout is an extremely rare condition with few case reports in the literature. It is an important differential diagnosis because the treatment depends on targeted therapy, unlike the main causes of sesamoiditis that often involves immobilization with special orthoses and prescription of anti-inflammatory drugs. We report here a case of a 38-year-old male, athlete, with bipartite medial sesamoid, who had insidious pain in the base of the left hallux. Laboratory tests showed no alterations, and imaging examinations demonstrated sesamoiditis with suspicion of stress fracture. The patient was initially prescribed an immobilization boot and analgesic and anti-inflammatory drugs, but he did not respond to the measures taken. After the onset of the same condition in the contralateral foot and getting the same imaging findings, we began an investigation of systemic disease, focusing on gout, because of a positive family history, which was confirmed by dual-energy computed tomography.

## 1. Introduction

Bone pain in the sesamoids can be caused by different groups of pathologies and usually results in substantial physical limitation, especially in athletes, who make up the population at greatest risk for developing the condition [1, 2]. Differential diagnosis is complex, involving all the diseases that affect the structures of the complex that includes the hallux and the metatarsophalangeal joint, and the investigation is usually expensive with respect to both time and resources. Among the pathologies that affect the sesamoids and cause local pain, most notable is trauma with subsequent fracture (mainly stress fracture) and development of local inflammation (sesamoiditis), which is usually due to intensive physical activity. In addition, there is also congenital or surgical absence of the sesamoid bones, infections, sesamoiditis of other etiologies, symptomatic partite sesamoid, osteochondritis, local nerve compression, and avascular necrosis of the sesamoid bones, which are also usually related to physical effort [2, 3].

Regarding sesamoiditis, its definition is varied, but it basically consists of any inflammatory condition that affects the sesamoid bones and causes symptoms [4]. Its causes include traumatic stress related to repetitive physical activity, infections, osteoarthritis, avascular necrosis, and inflammatory arthropathies [2, 5], with few case reports in the literature of sesamoiditis secondary to gout [6–9].

Gout is a systemic inflammatory disease of uric acid metabolism characterized by the deposition of sodium monourate crystals in soft tissues and joints [10]. Patients with high serum uric acid levels are more predisposed to the development of gout and its crises, although during crises, uric acid levels may be normal [10]. The metatarsophalangeal joint is the most affected [10], with rare reports demonstrating isolated clinical involvement of the sesamoid bone [6–9]. The main risk factors for developing the disease are male gender, positive family history, hypertension, alcohol consumption, excessive protein consumption, age, obesity, renal failure, and use of diuretics [10].

We present here a case report of bilateral sesamoiditis as the first manifestation of gout in a male athlete. It is a rare cause of pain in the sesamoids, which is usually related to stress injury secondary to physical activity.

## 2. Case Report

**Figure 1:**
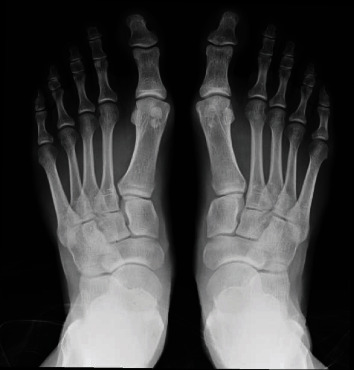
X-ray of the right and left feet demonstrating bilateral bipartite sesamoid bone and absence of other alterations.

**Figure 2:**
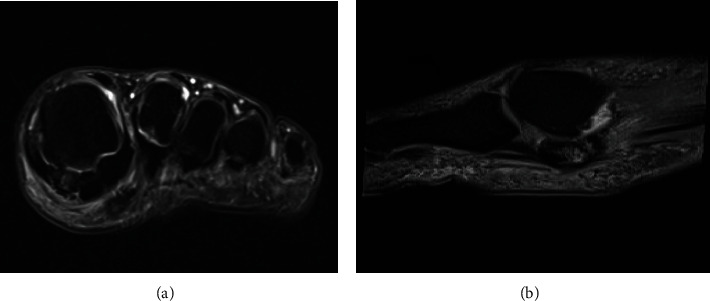
Magnetic resonance imaging of the left foot demonstrating bipartite medial sesamoid, diffuse homogeneous bone marrow edema of the medial sesamoid, periarticular soft tissue edema, and small glenosesamoid joint effusion ((a) coronal T2 with fat saturation, (b) sagittal T2 with fat saturation).

A 38-year-old white male patient started experiencing daily pain of high intensity and mechanical characteristics on the medial plantar surface at the base of the left hallux in October 2018. Associated with this, he had self-limiting episodes of slight local swelling without related inflammatory signs. The patient was an athlete who participated in daily physical activity (soccer and tennis) and who needed to stop due to substantial limitation because of pain. He did not have chronic comorbidities and did not take medications. He drank alcohol in small/moderate amounts on weekends, was not a smoker, and had a positive family history of gout (father). On general physical examination, there were no alterations, except for limited dorsiflexion of the left hallux and discrete local swelling. As a result of the above, he was submitted to preliminary laboratory tests and imaging examinations. The laboratory tests showed the following results: hemoglobin, 14.4 g/dL; leukocytes, 5570 cells/mm3; platelets, 208,000 cells/mm3; uric acid, 5.6 mg/dL; erythrocyte sedimentation rate, 11 mm; CRP, negative; antinuclear factor, negative; rheumatoid factor, 5.3 U/mL; creatinine, 0.8 mg/dL; urea, 48 mg/dL; fasting glucose, 82 mg/dL; A1C, 5.2%; sodium, 139 mEq/L; potassium, 4.0 mEq/L; AST, 16 IU/L; ALT, 20 IU/L; triglycerides, 99 mg/dL; total cholesterol, 171 mg/dL; LDL cholesterol, 109 mg/dL; HDL cholesterol, 43 mg/dL; urinalysis, normal; and 24-hour urinary uric acid, 639 mg. Complementary investigation with foot X-ray showed only bilateral bipartite medial sesamoid (Figure 1), while magnetic resonance imaging (MRI) of the left foot demonstrated bipartite medial sesamoid with suspected stress fracture, diffuse marrow edema of the medial sesamoid, periarticular soft tissue edema, and small joint effusion (Figure 2).

**Figure 3:**
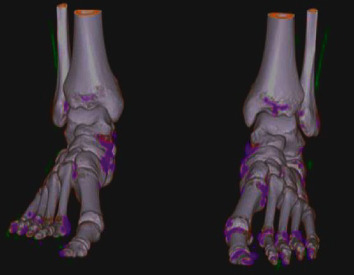
Dual-energy CT demonstrating sodium monourate deposits in both feet.

It was concluded that it was a case of stress-related sesamoiditis with possible associated fracture in the bipartite medial sesamoid, and we decided on clinical treatment with the use of an orthopedic boot for local immobilization, anti-inflammatory drugs (nonsteroid anti-inflammatory drug and intramuscular betamethasone) and a nonnarcotic analgesic (dipyrone). There was partial improvement and subsequent recurrence. After 4 months, he was showing the same picture in the contralateral foot, which prompted us to deepen our investigation of systemic disease that causes joint involvement, with an emphasis on gout. Thus, due to the impossibility of joint puncture due to the absence of significant effusion, dual-energy computed tomography (CT) was performed, which identified deposits of sodium monourate crystals in several structures of the feet (Figure 3), which led to the diagnosis of gout. Colchicine treatment was started, which led to the resolution of symptoms in one week, and later, chronic therapy with allopurinol was introduced. After 2 months, the patient was asymptomatic and resumed all his sports activities at the same intensity as before.

## 3. Discussion

We describe a case of bilateral medial sesamoiditis as the first manifestation of gout in a male athlete with a positive family history of the disease, who was treated for 4 months as a patient with stress-related sesamoiditis with a possible associated fracture in a bipartite medial sesamoid.

The clinical picture of sesamoiditis is characterized by pain in the medial plantar surface of the affected base of the hallux, whose characteristics depend on the etiology of the inflammatory process, but which in most cases is usually continuous, progressive, and initially insidious. Associated with this, pain worsens on the dorsiflexion of the hallux, and on physical examination, there was limitation of movement, local swelling, and pain on palpation in the sesamoid region [2]. In cases secondary to gout, pain manifestations are similar to those described here [6].

The diagnosis of sesamoiditis and its etiological process depend on complementary tests, especially imaging. Radiographic evaluation is important and may demonstrate structural alterations of sesamoid bones secondary to chronic inflammation, such as bone sclerosis and areas of fragmentation, as well as demonstrating the causative factor, such as fractures and osteoarthritis [2, 11]. However, radiography is sometimes normal and other examinations become necessary, particularly MRI, which shows diffuse bone marrow edema (hyposignal on T1- and hypersignal on T2-weighted images with fat saturation) and variable degree of joint or periarticular effusion, as well as determining the pathogenesis of the process, such as stress fractures, avascular necrosis, and other pathologies [2, 12]. In cases secondary to gout, traditional preliminary examinations are usually not sufficient for diagnosis, so laboratory tests and other resources should be used, such as joint puncture (if there is joint involvement), dual-energy CT, and even bone biopsy [10].

Regarding dual-energy CT in gout diagnosis, the exam is considered positive by the presence of color-coded monosodium urate (MSU) at joints, and periarticular spaces and its sensitivity and specificity are 84%–90% and 83%–93%, respectively [13, 14]. The detection of MSU crystals in the symptomatic joints on dual CT scan is sufficient for the diagnosis of gout. This is based on the Study for Updated Gout Classification Criteria (SUGAR) and includes clinical, laboratory, and imaging characteristics of gout [15, 16]. The dual-energy CT scan has several advantages, including its capacity to avoid an invasive procedure like synovial fluid aspiration, which carries the risk of bleeding and infection and has low yield in small joints. Regarding its mains limitations, it is user dependent, and imaging results should be interpreted with caution by a professional who is well trained to recognize artifacts. In addition, false positive results are also observed in patients with high-grade osteoarthritis [13, 17, 18].

The approach of sesamoiditis depends on its etiology but most often involves immobilization with special orthoses and prescription of anti-inflammatory drugs, since its main cause is local inflammation due to bone stress injury. In some cases, surgical treatment is necessary because of the refractoriness of the conservative approach [19]. However, in special situations, there is targeted therapy, and the success of the approach depends on the correct diagnosis. In sesamoiditis secondary to gout, the treatment of the disease usually leads to substantial improvement, where surgical procedures are reserved for patients with advanced and destructive lesions [6–9].

In our case report, a 38-year-old male patient, athlete, with bipartite medial sesamoid, developed insidious pain in the medial plantar surface at the base of the left hallux. After laboratory tests showed no alterations and after imaging demonstrated sesamoiditis with suspicion of stress fracture, this case was first approached as a classic condition but without response to the measures taken. Subsequent to the onset of the same condition in the contralateral foot and getting the same imaging findings, we began our investigation of systemic disease, focusing on gout, due to a positive family history. Gout was confirmed with dual-energy CT and treatment with colchicine and allopurinol was begun, which resulted in the resolution of clinical symptoms. These findings are in agreement with previous publications, especially the study by Mair et al., who described a case of medial sesamoiditis on the right secondary to gout in an 18-year-old male athlete with a bipartite sesamoid but without a diagnosis of gout (initial uric acid of 7.5 mg/dL), but who had a definitive diagnosis of the disease after histopathological analysis of sesamoidectomy material [6]. In addition, Wakhlu reported a case of a 45-year-old male patient who developed bilateral sesamoiditis secondary to gout [8], and Balutis and coworkers reported a 37-year-old male patient with normal uric acid levels who developed an extensive lesion in the left sesamoid defined as gout in the histological evaluation of the excised lesion [9].

## References

[1] McBryde A. J., Anderson R. B. (1988). Sesamoid foot problems in the athlete. *Clinics in Sports Medicine*.

[2] Sanders T. G., Rathur S. K. (2008). Imaging of painful conditions of the hallucal sesamoid complex and plantar capsular structures of the first metatarsophalangeal joint. *Radiologic Clinics of North America*.

[3] Biedert R. (1993). Which investigations are required in stress fracture of the great toe sesamoids?. *Archives of Orthopaedic and Trauma Surgery*.

[4] Karasick D., Schweitzer M. E. (1998). Disorders of the hallux sesamoid complex: MR features. *Skeletal Radiology*.

[5] Perlman P. (1994). First metatarsal sesamoid pain. *Australian Podiatrist*.

[6] Mair S. D., Coogan A. C., Speer K. P., Hall R. L. (1995). Gout as a source of sesamoid pain. *Foot & Ankle International*.

[7] Reber P. U., Patel A. G., Noesberger B. (2016). Gout: rare cause of hallucal sesamoid pain: a case report. *Foot & Ankle International*.

[8] Wakhlu A. (2004). An uncommon cause of great toe pain: sesamoiditis. *Journal of Indian Rheumatology Association*.

[9] Balutis E., Pino A. (2015). Gout causing isolated sesamoid destruction mimicking a neoplastic process. *American Journal of Orthopedics*.

[10] Ragab G., Elshahaly M., Bardin T. (2017). Gout: an old disease in new perspective – a review. *Journal of Advanced Research*.

[11] Mittlmeier T., Haar P. (2004). Sesamoid and toe fractures. *Injury*.

[12] Srinivasan R. (2016). The hallucal-sesamoid complex: normal anatomy, imaging, and pathology. *Seminars in Musculoskeletal Radiology*.

[13] Bongartz T., Glazebrook K. N., Kavros S. J. (2015). Dual-energy CT for the diagnosis of gout: an accuracy and diagnostic yield study. *Annals of the Rheumatic Diseases*.

[14] Choi H. K., Burns L. C., Shojania K. (2012). Dual energy CT in gout: a prospective validation study. *Annals of the Rheumatic Diseases*.

[15] Neogi T., Jansen T. L., Dalbeth N. (2015). 2015 gout classification criteria: an American College of Rheumatology/European League Against Rheumatism collaborative initiative. *Annals of the Rheumatic Diseases*.

[16] Taylor W. J., Fransen J., Jansen T. L. (2015). Study for updated gout classification criteria: identification of features to classify gout. *Arthritis Care and Research*.

[17] Monu J. U., Pope T. L. (2004). Gout: a clinical and radiologic review. *Radiologic Clinics of North America*.

[18] Glazebrook K. N., Guimarães L. S., Murthy N. S. (2011). Identification of intraarticular and periarticular uric acid crystals with dual-energy CT: initial evaluation. *Radiology*.

[19] Schein A. J., Skalski M. R., Patel D. B. (2015). Turf toe and sesamoiditis: what the radiologist needs to know. *Clinical Imaging*.

